# Supplementary material for: 2D Pose Estimation vs. Inertial Measurement Unit-Based Motion Capture in Ergonomics: Assessing Postural Risk in Dental Assistants
Source: Bioengineering (Basel). 2025 Apr 10;12(4):403. doi: 10.3390/bioengineering12040403 (PMC12024670; doi:10.3390/bioengineering12040403)
Supplement: Supplementary file 1 [file bioengineering-12-00403-s001.zip › bioengineering-3539917-supplementary.pdf]

Supplementary material

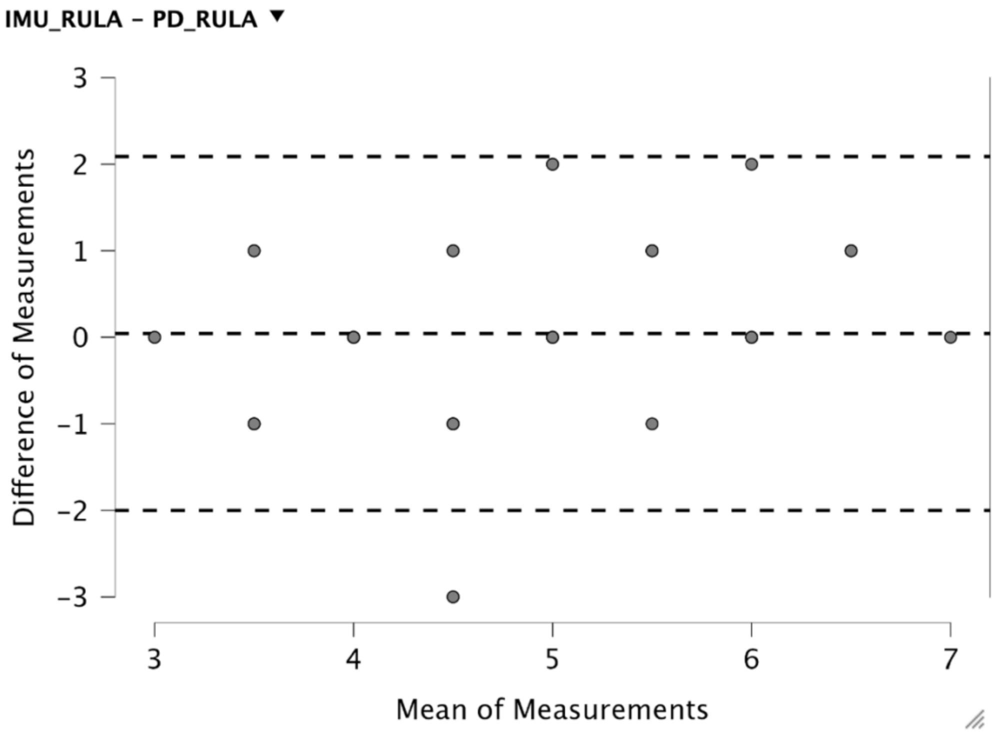

Figure S1. Scatter plot representing overall RULA-scores.

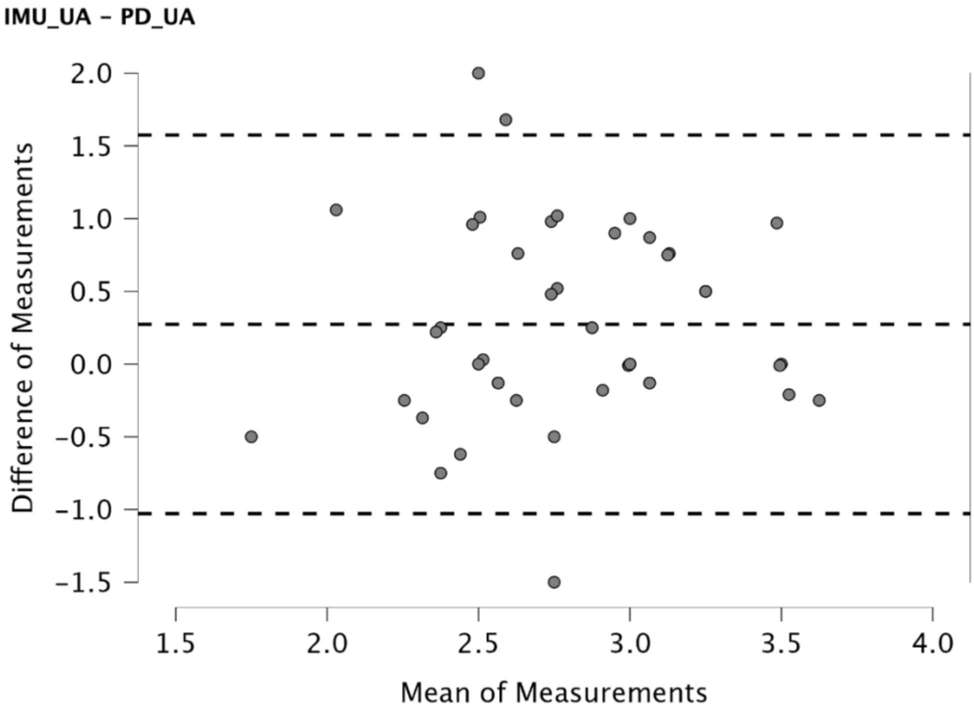

Figure S2. Scatter plot representing upper arm RULA-part-scores.

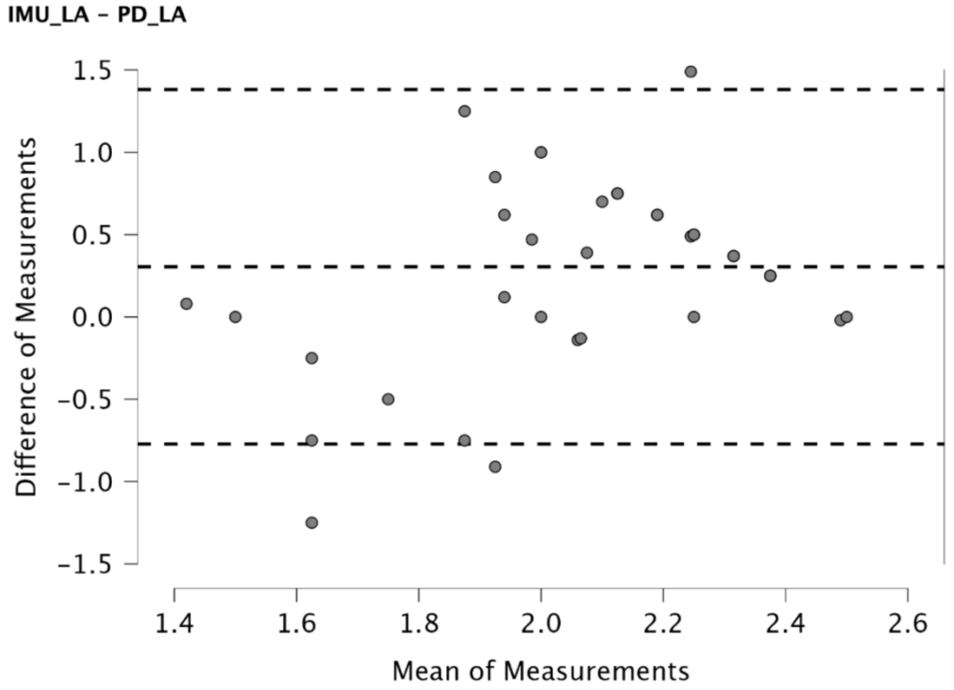

Figure S3. Scatter plot representing lower arm RULA-part-scores.

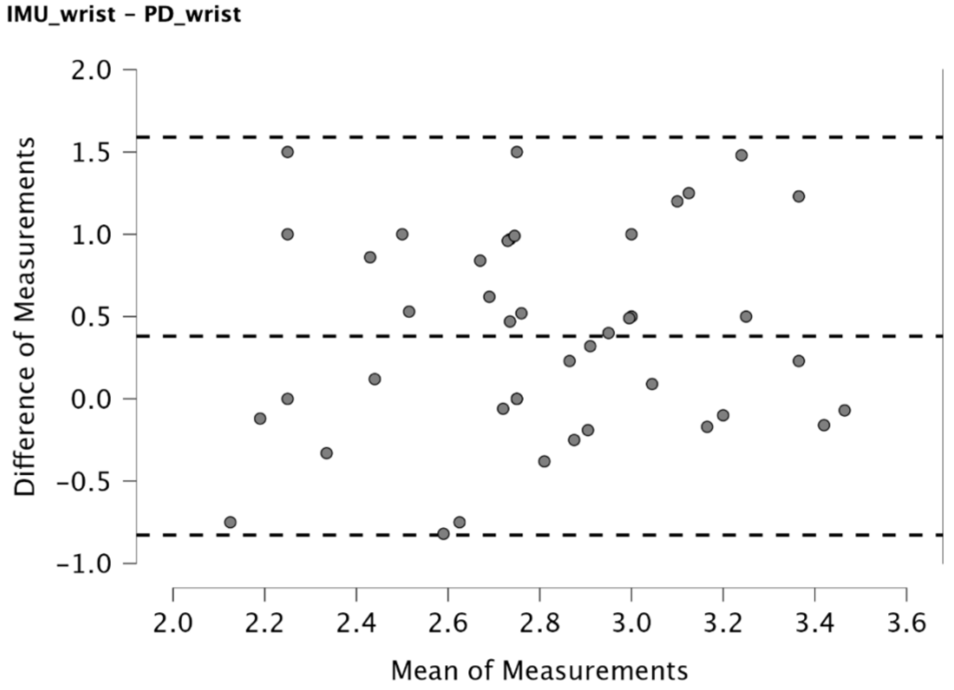

Figure S4. Scatter plot representing wrist RULA-part-scores.

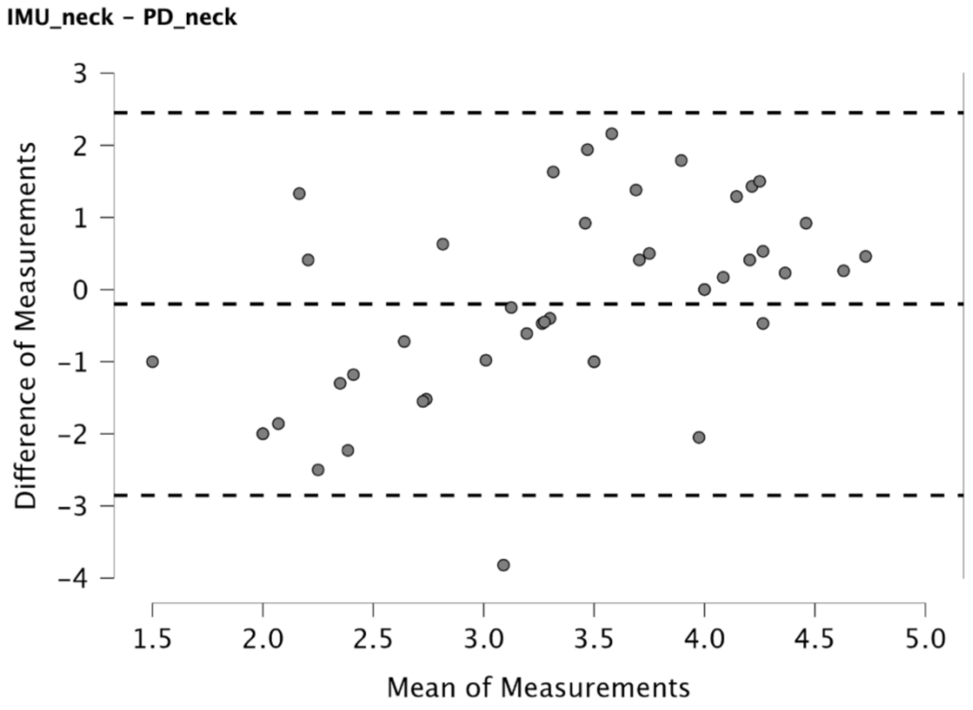

Figure S5. Scatter plot representing neck RULA-part-scores.

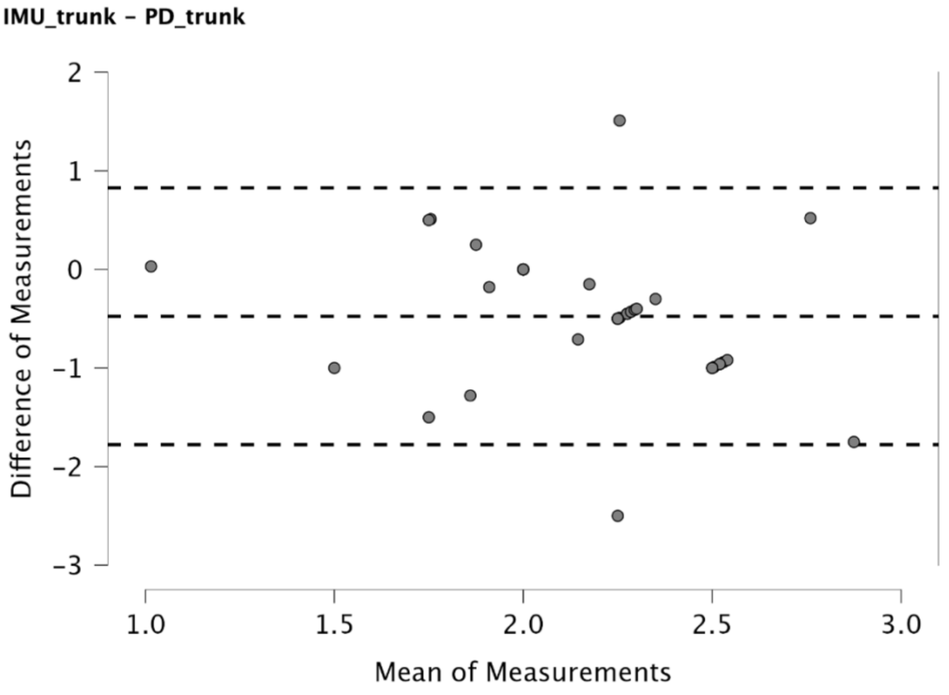

Figure S6. Scatter plot representing trunk RULA-part-scores.
